# Gastro-Oesophageal Reflux in Noncystic Fibrosis Bronchiectasis

**DOI:** 10.1155/2011/395020

**Published:** 2011-11-10

**Authors:** Annemarie L. Lee, Brenda M. Button, Linda Denehy, John W. Wilson

**Affiliations:** ^1^Department of Physiotherapy, Melbourne School of Health Sciences, The University of Melbourne, Melbourne, VIC 3010, Australia; ^2^Department of Physiotherapy, The Alfred Hospital, Commercial Road, Melbourne, VIC 3004, Australia; ^3^Department of Medicine, Monash University, Melbourne, VIC 3088, Australia; ^4^Department of Allergy, Immunology and Respiratory Medicine, The Alfred Hospital, Commercial Road, Melbourne, VIC 3004, Australia

## Abstract

The clinical presentation of noncystic fibrosis bronchiectasis may be complicated by concomitant conditions, including gastro-oesophageal reflux (GOR). Increased acidic GOR is principally caused by gastro-oesophageal junction incompetence and may arise from lower oesophageal sphincter hypotension, including transient relaxations, hiatus hernia, and oesophageal dysmotility. Specific pathophysiological features which are characteristic of respiratory diseases including coughing may further increase the risk of GOR in bronchiectasis. Reflux may impact on lung disease severity by two mechanisms, reflex bronchoconstriction and pulmonary microaspiration. Symptomatic and clinically silent reflux has been detected in bronchiectasis, with the prevalence of 26 to 75%. The cause and effect relationship has not been established, but preliminary reports suggest that GOR may influence the severity of bronchiectasis. Further studies examining the implications of GOR in this condition, including its effect across the disease spectrum using a combination of diagnostic tools, will clarify the clinical significance of this comorbidity.

## 1. Introduction

Bronchiectasis which is unrelated to cystic fibrosis (noncystic fibrosis bronchiectasis) is a chronic and progressive respiratory disease, associated with irreversible, abnormal dilation of the bronchi and bronchioles. There are a multitude of aetiologies, ranging from idiopathic to congenital conditions, immunological disorders and postinfective causes [1]. The spectrum of bronchiectasis is predominantly characterised by chronic cough, sputum production, dyspnoea, and fatigue [2, 3]. The clinical course is generally punctuated by infectious exacerbations [1] and together, these features negatively impact on health-related quality of life in bronchiectasis [4, 5]. 

Although the prevalence of bronchiectasis in patients secondary to recurrent or severe infection has declined in recent years [3, 6], it remains problematic and is associated with significant morbidity and mortality [6, 7]. A recent study demonstrated persistent and progressive respiratory symptoms and concurrent decline in lung function despite ongoing medical intervention over an eight-year period [7]. The goals of management are multifaceted, aiming to minimise the frequency and severity of exacerbations, rate of pulmonary decline, and to maximise secretion clearance. This is achieved through antibiotic therapy, inhalation therapy, and physiotherapy [1, 8]. 

The clinical presentation of bronchiectasis may be complicated by the coexistence of other medical conditions or comorbidities, including gastro-oesophageal reflux (GOR) [9]. GOR refers to the regurgitation of gastric contents into the oesophagus, with 24 hr oesophageal pH monitoring providing a comprehensive quantification of GOR in the distal and proximal oesophagus [10]. A comorbidity such as GOR may reduce health-related quality of life and accelerate the rate of pulmonary decline and progression of bronchiectasis [1]. Understanding the extent of GOR and the clinical relevance of a concomitant diagnosis in bronchiectasis is important in the overall management of this condition.

## 2. Pathological Reflux

In defining GOR, it is important to acknowledge that the process of occasional reflux of gastric contents into the oesophagus is a normal physiological event [11]. Generally, such episodes occur five times during the postprandial hour, with their frequency declining rapidly to a baseline of zero approximately one to two hours after post prandial [11]. Most episodes are restricted to the distal oesophagus, are of brief duration, cleared rapidly, and generally well tolerated [12]. In contrast, pathological GOR has been described as the increased frequency or duration of exposure of the oesophagus to regurgitated gastric contents [13]. The frequency and duration of episodes, as well as the volume, composition, and destination of the gastro-oesophageal refluxate are all factors determining its significance. 

Dysfunction of the oesophago-gastric junction is a prerequisite for the development of GOR. The competence of this barrier is the product of its anatomical and physiological features [14]. This antireflux barrier is dynamic and is required to provide protection from reflux during different physiological circumstances [12]. When the aggressive forces (acid reflux) outweigh the defensive forces (antireflux barrier and oesophageal clearance), the end result is GOR. The intermittent nature of GOR in some individuals suggests that these forces are delicately balanced. 

The aetiology of GOR is multifactorial and includes gastro-oesophageal junction incompetence, characterised by transient lower oesophageal sphincter (LOS) relaxation, hypotensive LOS, and hiatus hernia [15–17]. There are factors specific to pulmonary diseases which may also contribute to the development or occurrence of GOR, including physiological changes in respiratory mechanics. During inspiration, a raised intraabdominal pressure increases the risk of GOR [16]. Both airflow obstruction and hyperinflation are believed to interfere with the diaphragmatic crural support augmenting LOS pressure [18], although the possible contribution of respiratory mechanics to GOR in chronic lung disease remains unclear [16, 17, 19]. 

Chronic reduction in LOS tone can also be associated with GOR by the mechanism of stress reflux, where a hypotensive LOS is overcome by an abrupt increase in intraabdominal pressure, such as during coughing [15, 16]. The temporal association between coughing and episodes of reflux has been demonstrated in patients with chronic cough and asthma [20, 21], which suggests that a self-perpetuating positive feedback cycle of cough stimulating reflux may occur in patients with preexisting pulmonary disease [21]. Other potential factors which may influence GOR include oesophageal acid clearance. Oesophageal acid clearance is dependent on effective oesophageal emptying. Prolongation of acid clearance secondary to oesophageal dysmotility, with low amplitude oesophageal contractions and abnormal peristalsis, has been documented in patients with asthma and chronic bronchitis [22, 23]. 

There are two principle mechanisms by which GOR may impact on respiratory disease severity. The first is vagally mediated reflex bronchoconstriction [24]. Oesophageal acid in the distal oesophagus stimulates airway irritation and an inflammatory response, with the release of potent mediators of bronchoconstriction [25], which are associated with coughing and reflux-induced bronchoconstriction in patients with asthma [21]. The second mechanism is pulmonary microaspiration. During microaspiration, refluxed gastric material reaches the proximal oesophagus and moves into the hypopharynx with the potential to enter the larynx or the trachea [24]. 

In addition to these pathophysiological mechanisms, aspects of physiotherapy management of bronchiectasis may influence the frequency of GOR. Previous studies have demonstrated that airway clearance techniques to facilitate sputum expectoration involving gravity-assisted drainage with a head down tilt provoke GOR in patients with cystic fibrosis [26]. This is a type of therapy regularly prescribed by physiotherapists for individuals with bronchiectasis [27], although the impact of this airway clearance technique on the frequency of reflux episodes in this population has not been studied. 

The interaction between chronic respiratory diseases and GOR is not yet fully defined, nor has cause and effect been clearly established. Similarly, the precise nature of the relationship between GOR and bronchiectasis remains elusive. Some individuals may have two independent conditions which merely coexist; in others, a concomitant diagnosis of GOR may have significant clinical implications. In view of this, the diagnostic approach to GOR in patients with chronic respiratory conditions, including bronchiectasis may require careful consideration, with an accurate diagnosis remaining a clinical challenge.

## 3. Diagnostic Tools

Comprehensive symptom evaluation is important with weekly experience of heartburn and/or acid regurgitation considered a strong diagnostic indicator of GOR [28]. However, symptom questionnaires focus on the presence of gastrointestinal symptoms alone, which limits their application to patients with occult or clinically silent GOR. Even with the recognition that respiratory manifestations, including cough, wheezing, and dyspnoea may be an atypical presentation of GOR beyond the oesophagus [29, 30], these symptoms are common amongst patients with bronchiectasis, and their ability to distinguish a diagnosis of GOR has not been fully determined. 

Ambulatory intraoesophageal pH monitoring is performed with a pH probe passed nasally and positioned 5 cm above the LOS and this detects reflux episodes by a drop in pH to less than 4 [31]. The majority of the literature indicates that ambulatory 24 hr oesophageal pH monitoring yields the highest sensitivity and specificity of all diagnostic tests of GOR with up to 98% and 96%, respectively, [32]. Dual-channel oesophageal pH monitoring allows the recording of proximal oesophageal reflux [33]. The importance of nonacid, liquid, and a mixture of gas and liquid reflux has been highlighted with the application of multichannel intraluminal impedance monitoring, which allows recording of GOR at all pH levels and measurement of the air-liquid composition of the refluxate [34]. This is emerging as a useful tool for diagnosing nonacid and weakly acidic refluxate [35, 36] and has been applied in other respiratory conditions, including cystic fibrosis [37, 38]. It is yet to be used in the bronchiectasis population.

Detection of pulmonary microaspiration is even more difficult. Nuclear scintography is used as a semiquantitative test for detection of microaspiration secondary to GOR, but the small volume required to induce bronchospasm may escape detection or may occur intermittently [39]. Recent studies have explored noninvasive methods of measuring microaspiration. The detection of pepsin as a surrogate marker of GOR has been examined using pepsin assays in sputum and saliva samples, yielding a sensitivity and specificity of 75% and 91%, respectively, compared to proximal pH monitoring [40]. More recent studies have tested bronchoalveolar lavage fluid for pepsin and bile salts, providing evidence of aspiration of gastric contents into the lower respiratory tract in patients with cystic fibrosis and lung transplant recipients [37, 38, 41, 42]. Another method is exhaled breath condensate. Exhaled breath condensate provides a noninvasive quantification of endogenous airway pH, with low pH levels in exhaled breath condensate demonstrated in patients with stable respiratory disease, including asthma, cystic fibrosis, chronic obstructive pulmonary disease, and bronchiectasis [43, 44]. While the clinical utility of exhaled breath condensate in the diagnosis of pulmonary microaspiration has not been fully determined in lung disease, it is a promising noninvasive option.

## 4. GOR in Bronchiectasis

Amongst the disparate aetiologies of bronchiectasis, it has been hypothesised that GOR is a potential cause of this condition, with aspiration into the tracheobronchial tree hypothesised to present as insidious-onset bronchiectasis, via gastric acid-induced erosion triggering chronic airway inflammation [3, 45]. Helicobacter pylori (HP) has been identified as the microorganism which causes gastritis [46], and a high seroprevalence (76%) of HP in patients with bronchiectasis has been detected [47]. It has been proposed that inhalation or aspiration of HP exotoxins may contribute to chronic airway inflammation in patients with idiopathic bronchiectasis [47]. However, in the same cohort of patients, symptoms of heartburn and acid regurgitation were not linked with high levels of serum HP, and concurrent oesophageal pH monitoring was not included to confirm a diagnosis of GOR [9]. A later study failed to demonstrate HP in bronchial specimens in patients with bronchiectasis arising from a variety of aetiologies [48]. With these disparate results, the possible pathogenic role of HP in bronchiectasis requires further study. 

Several studies have explored the causal relationship between GOR and bronchiectasis in the paediatric population with confirmed bronchiectasis. Using a mix of diagnostic tools, including distal channel oesophageal pH monitoring, barium oesophagogram and radiological findings, these reviews suggested that GOR is the causative factor in 3% to 32% of paediatric patients with bronchiectasis [49, 50]. 

GOR is also a suggested cause of idiopathic bronchiectasis in the adult population, with the presence of upper gastrointestinal symptoms, including epigastric pain, abdominal distention, vomiting, heartburn, and acid regurgitation identified in 32% out of a cohort of 100 patients, 82% of whom had idiopathic bronchiectasis [9, 47]. In an equally large study of 100 adults with bronchiectasis, GOR was identified as a causative factor in 3% of patients, based on gastrointestinal symptoms and symptomatic improvement following commencement of antireflux medication [51]. Shoemark and colleagues [52] found aspiration to be a cause of bronchiectasis in 1% of patients. While these reports suggest a degree of causality, the lack of objective diagnostic confirmation of GOR implies that further clarification is necessary in patients with idiopathic bronchiectasis using an objective measurement of GOR. 

Asymptomatic (clinically silent) nocturnal GOR was diagnosed in a group of 25 patients with a range of respiratory conditions, including bronchiectasis, using a barium oesophagogram, with only 40% of patients reporting heartburn and 16% reporting dysphagia [45]. This study was one of the first to highlight the significance of objective tools to confirm a diagnosis of GOR. Since this early report, a further five studies have evaluated the prevalence of GOR and its clinical significance in patients with bronchiectasis, using a combination of symptomatic and objective tools. Symptomatic evaluation found that 32% of 100 patients with bronchiectasis experienced epigastric pain, abdominal distension, vomiting, heartburn, or acid regurgitation [9]. The significance of these symptoms is highlighted by the reduced lung function associated with acid regurgitation and the link between epigastric pain and a higher number of lobes affected by bronchiectasis [9]. While this provided a glimpse into the potential impact of GOR on respiratory function in bronchiectasis, these findings remain limited to those with typical GOR symptoms. 

Four studies have examined GOR in bronchiectasis using 24 hr oesophageal pH monitoring. A pilot study of 19 patients with bronchiectasis utilized simultaneous tracheal and oesophageal pH monitoring [53]. A total of eight patients demonstrated reflux based on the DeMeester score [53]. Of those with GOR, 88% experienced symptoms of heartburn, nocturnal cough, or disturbed sleep compared to those without reflux. While the results are suggestive that a proportion of patients with bronchiectasis have distal reflux with associated nocturnal symptoms, no microaspiration of gastric contents was demonstrated with tracheal monitoring [53]. 

In four patients with end-stage bronchiectasis, who completed dual-channel oesophageal pH monitoring, the prevalence of distal reflux of 75% and proximal reflux of 50% suggests that patients with more severe bronchiectasis may be more likely to have GOR [54]. However, the relationship of reflux to clinical presentation or its association with lung disease severity was not evaluated [54]. Similar results were found in a study of seven patients with advanced bronchiectasis awaiting lung transplantation, with 33% experiencing an increased number of distal reflux episodes, specifically in the supine position [55]. In addition, manometry studies showed LOS hypotonia in 57% of those patients with upper oesophageal sphincter hypotonia in 14% of patients. This suggests that the gastrointestinal contributions to GOR which have been identified in patients with chronic obstructive pulmonary disease, cystic fibrosis, and interstitial lung disease [54, 55] may also be apparent in bronchiectasis. 

In the largest prospective study to date of a group of 58 patients with nontuberculous mycobacterium lung disease with associated bronchiectasis, GOR was diagnosed in 26%, with clinically silent reflux in 73% [56]. Neither the presence of typical symptoms of heartburn nor acid regurgitation were predictive of GOR. However, those with GOR demonstrated more extensive bronchiectasis on high resolution computed tomography and bronchiolitis, with more lobes affected compared to those without GOR [56], which illustrates the potential clinical implications of GOR. 

These studies suggest that GOR is a comorbidity in selected patients with bronchiectasis, and the reported prevalence is currently higher compared to the general population (range of 10 to 20%) [57]. While the understanding of GOR and its relationship to bronchiectasis have been significantly enhanced by studies using oesophageal pH monitoring, it is evident that the clinical presentation may not include typical symptoms of GOR, emphasising the ongoing value of objective evaluation. GOR is associated with a decreased health-related quality of life in individuals without lung disease who have symptoms of heartburn and acid regurgitation [58], but the degree of compromise to health-related quality of life in patients with bronchiectasis has not been examined. These clinical consequences are worthy of future study.

## 5. Current Therapy and Management

Current therapy for GOR focuses on modifying risk factors, inhibiting the production of gastric acid and enhancing oesophageal and gastric motility. Recommended lifestyle changes to reduce the incidence of reflux include avoidance of potential dietary triggers and meals approximately two hours before recumbence with elevation of the head of the bed for those with nocturnal GOR [59]. Medical approaches include the use of Antacids, Histamine antagonists, and Proton pump inhibitors. The beneficial effect of Antacids is related to the neutralization of acid, which provides temporary symptomatic relief [60]. Proton pump inhibitors and Histamine antagonists inhibit gastric acid secretion, with Proton pump inhibitors being the more effective agent [60]. In the absence of amelioration in symptoms or persistence of the underlying mechanism causing GOR, surgery is an additional option. The direct effects of these approaches in patients with bronchiectasis are unknown. With respect to the surgical management, fundoplication in two patients with advanced bronchiectasis was associated with improvement in lung function and reduction in oxygen requirements [61, 62]. Further exploration of the management of GOR in bronchiectasis is required.

## 6. Conclusions

Although the prevalence of bronchiectasis has declined in recent years, a low index of clinical suspicion persists with this disease [63]. Regardless of the source of bronchiectasis, the possible contribution of GOR to rate of decline in lung function requires further evaluation. In addition, the symptoms of GOR in patients with bronchiectasis in differing clinical states are unclear. With acute exacerbations accompanying the clinical course of this condition, it would be worthy to note the pattern and frequency of GOR episodes in patients who are acutely unwell. This could provide insight into the possible contribution of this comorbidity to the progression and prognosis of bronchiectasis. The pathogenesis and features of GOR in patients with bronchiectasis are complex and the application of a range of reliable diagnostic tests to identify the extent of the comorbidity and its specific features, including acidic, weakly acidic, non-acid or duodenogastric reflux, and pulmonary microaspiration are important. Further work is required to identify any specific gastro-oesophageal features or respiratory mechanics which may heighten the risk of GOR in patients with bronchiectasis. Finally, the optimal treatment approaches for managing GOR in this population are yet to be established.
